# Trends in parkinson’s disease mortality in China from 2004 to 2021: a joinpoint analysis

**DOI:** 10.1186/s12889-024-18532-8

**Published:** 2024-04-19

**Authors:** Suxian Wang, Shuai Jiang, Jian Wu, Yudong Miao, Yanran Duan, Zihan Mu, Jing Wang, Yanyu Tang, Mingzhu Su, Zixu Guo, Xueqing Yu, Yaojun Zhao

**Affiliations:** 1 School of Public Health, Zhengzhou University, 450001 Zhengzhou, Henan Province China; 2 The First Affiliated Hospital of Zhengzhou University, 450052 Zhengzhou, Henan Province China; 3 Institute for Hospital Management of Henan Province, 450052 Zhengzhou, Henan Province China; 4 Central China Fuwai Hospital, Central China Fuwai Hospital of Zhengzhou University, 451460 Zhengzhou, Henan Province China

**Keywords:** Mortality, Parkinson's disease, Trend analysis, Joinpoint regression

## Abstract

**Background:**

This study aimed to analyze the trends of Parkinson’s disease (PD) mortality rates among Chinese residents from 2004 to 2021, provide evidence for the formulation of PD prevention and control strategies to improve the quality of life among PD residents.

**Methods:**

Demographic and sociological data such as gender, urban or rural residency and age were obtained from the National Cause of Death Surveillance Dataset from 2004 to 2021. We then analyzed the trends of PD mortality rates by Joinpoint regression.

**Results:**

The PD mortality and standardized mortality rates in China showed an overall increasing trend during 2004–2021 (average annual percentage change [AAPC] = 7.14%, AAPC_ASMR_=3.21%, *P* < 0.001). The mortality and standardized mortality rate in male (AAPC = 7.65%, AAPC_ASMR_=3.18%, *P* < 0.001) were higher than that of female (AAPC = 7.03%, AAPC_ASMR_=3.09%, *P* < 0.001). The PD standardized mortality rates of urban (AAPC = 5.13%, AAPC_ASMR_=1.76%, *P* < 0.001) and rural (AAPC = 8.40%, AAPC_ASMR_=4.29%, *P* < 0.001) residents both increased gradually. In the age analysis, the mortality rate increased with age. And the mortality rates of those aged > 85 years was the highest. Considering gender, female aged > 85 years had the fastest mortality trend (annual percentage change [APC] = 5.69%, *P* < 0.001). Considering urban/rural, rural aged 80–84 years had the fastest mortality trend (APC = 6.68%, *P* < 0.001).

**Conclusions:**

The mortality rate of PD among Chinese residents increased from 2004 to 2021. Male sex, urban residence and age > 85 years were risk factors for PD-related death and should be the primary focus for PD prevention.

## Background

Parkinson’s disease (PD) is the second most common neurodegenerative disorder worldwide and is characterized by the loss of dopaminergic neurons in the substantia nigra and other brain regions [1–2]. Generally, neurological diseases are the leading cause of disability and the second leading cause of death worldwide, with PD being the fastest-growing [3]. Regions with a higher socioeconomic status such as Europe have surprisingly high rates of PD mortality compared to other countries, bringing heavy burdens to the individual and society [4]. In 2019, there were 8.51 million people with PD with a disability-adjusted life-year rate of 6.292, and 362,900 deaths were attributed to this condition [5]. By 2040, there are projected to be 12 million PD patients globally [6]. Since 1994, the mortality rate of PD has increased steadily. The mortality rate (per 100,000 persons) in 1994 was 1.76, which increased to 5.67 by 2019. The economic burden of PD was highest in those aged 70–84 years [4–7]. In 2019, there were approximately 2.84 million PD patients in China, accounting for 33.37% of global PD patients. China had the largest number of patients in any country, and the total disability-adjusted life years due to PD in China are increasing. With population growth and the increasing aging population, it is expected that the number of Chinese people with PD will increase to 4.94 million by 2030, which accounts for 57% of the global PD population [8]. The cause of PD is multi-factorial, where aging population, more accurate and earlier diagnosis, occupational and environmental exposure are the main reasons for the increase in the number of PD deaths globally [9–10]. People living in countries with higher socioeconomic status are more likely to go to the hospital for neurological clinic examination, and have more neurologists specializing in movement disorders to be diagnosed with PD [4].

Currently, PD research has primarily focused on its pathogenesis, diagnosis, treatment, and other medical techniques [11–13]. A previous study found a significant upward trend in PD mortality worldwide from 1994 to 2019 [4]. The mortality rate of PD increased between 1979 and 2009 in several European countries such as Iceland, Finland, Malta and Croatia [14]. A similar trend of increasing PD disease mortality was noted in the United States from 1999 to 2019 [10]. In contrast, scholars studied the mortality trend of PD in China from 1990 to 2019 using the Global Burden of Disease 2019, which showed a decreasing trend. And using the Bayesian age-period-cohort model predicted PD mortality will continue to decline from 2020–2030 [15]. A study found the age-standardized death rate of PD in China showed a downward trend from 1990 to 2019 [16]. According to the Chinese Guidelines for the Treatment of PD (4th edition), as the disease progresses, the motor and non-motor symptoms of PD will gradually worsen, impairing the patient’s daily activities and bringing a huge social and medical burden. In 2022, the Department of Aging Health issued a notice on comprehensively strengthening healthcare services for the elderly; guidelines were proposed to improve intervention strategies, enhance classification guidance for key chronic diseases of the elderly population and actively carry out early screening and provide health guidance for people affected by neurodegenerative diseases such as Alzheimer’s disease and PD. This study aimed to explore the trend of PD mortality by stratifying by gender, urban/rural residency and age in China based on a cause-of-death surveillance dataset from 2004 to 2021. Furthermore, our study may help develop effective prevention and control measures for PD and act as a scientific reference for devising public health policies.

## Method

### Data sources

PD classification code was G20–G21 according to the International Statistical Classification of Diseases and Related Health Problems 10th Revision. The mortality and standardized mortality rates of PD were classified according to sex, area (urban or rural) and age group. According to the China cause-of-death disease surveillance dataset, all counties (including county-level cities) in China were defined as rural, and all districts were defined as urban. Age subgroups were divided into 5-year intervals from 30 to 84 years, and > 85 years. Because aging was the most significant risk factor for developing PD and the mortality rate of PD under the age of 30 was zero, and mainly concentrated in the elderly in China.

The study data were derived from the Chinese cause-of-death surveillance dataset from 2004 to 2021 (https://ncncd.chinacdc.cn/xzzq_1/202101/t20210111223706.htm). The Chinese cause-of-death surveillance dataset dates back to 2004 and was published in 2021. Therefore, the period 2004–2021 was chosen as the timeline to study the changing trends in PD mortality. The national disease surveillance system was formally set up in 1980. According to the principle of multi-stage stratified cluster random sampling, the representative disease monitoring points were selected in 31 provinces have established a monitoring system in 1990, and built a new Disease Surveillance Points (DSP) system consisting of 145 monitoring points. In 2003, the DSP system was adjusted, and the monitoring sites were expanded to include 161 different points, accounting for approximately 6% of the country’s population. In 2013, the Chinese Government integrated and expanded the cause-of-death reporting system, establishing a provincial-representative national monitoring system incorporating 605 disease monitoring points that covered approximately 24% of the Chinese population.

### Quality control

In order to guarantee the high quality and credibility of monitoring system data, the data quality was evaluated before conducting the data analysis and forming the results; however, it was difficult to avoid omission. Consequently, some monitoring points that were considered serious omissions likely to affect the overall results were removed by filtering through the collected data and further comparing and judging the overall data quality of each monitoring point. From 2004 to 2012, we applied a mortality rate of less than 3‰ in each monitoring site as the exclusion criterion. The former Ministry of Health’s set mortality rate below 4.5‰ as the exclusion criterion between 2013 and 2021. Besides, the monitoring sites that were newly added in 2013 set a mortality rate of 5‰ as the exclusion criterion.

### Statistical analysis

Using the Joinpoint Regression software (Joinpoint Regression Program, National Cancer Institute, USA, *v*4.9.1.0), **w**e calculated the mortality and standardized mortality rates of PD. The number and location of connection points were judged by a Monte Carlo test based on a log-linear model with a Poisson distribution limited to a maximum of 1 Joinpoint. The annual percentage change (APC) and average annual percentage change (AAPC) with 95% confidence intervals (CIs) were used to describe the trends in mortality. The APC (1) and AAPC (2) were calculated as follows:

1 $${\rm{APC}} = \left\{ E \right.xp\left( {{b_i}} \right) - \left. 1 \right\} \times 100$$

2 $${\rm{AAPC}} = \left\{ e \right.xp\left( {\frac{{\mathop \sum \nolimits {w_i}{b_i}}}{{\mathop \sum \nolimits {w_i}}}} \right) - \left. 1 \right\} \times 100$$

where *b*_*i*_ is the slope i coefficient for each segment in the desired range of years, *w*_*i*_ refers to the length of each segment in the range of years segment in the desired range of years.

Data from the Fifth National Population Census (2000) were used to calculate the age-standardized mortality rate (ASMR) (3), which is calculated by using the age composition ratio of the same population (the standard population composition ratio) and actual age-specific mortality rate. ASMR are used to compare population-wide mortality rates in areas where there are differences in the age structure of two or more populations.

3 $${\rm{ASMR}} = \frac{{\mathop \sum \nolimits nPx*nMx}}{{\mathop \sum \nolimits nPx}}$$

where nP_*x*_ refers to the age-specific population of the standard population, nM_*x*_ refers to the age-specific mortality rate of the waiting standardized population, n is the spacing of each age group, and x is the starting age for each age group. All tests were two-sided, with *P* < 0.05 considered statistically significant.

## Results

### Trends of PD mortality

**Fig. 1:**
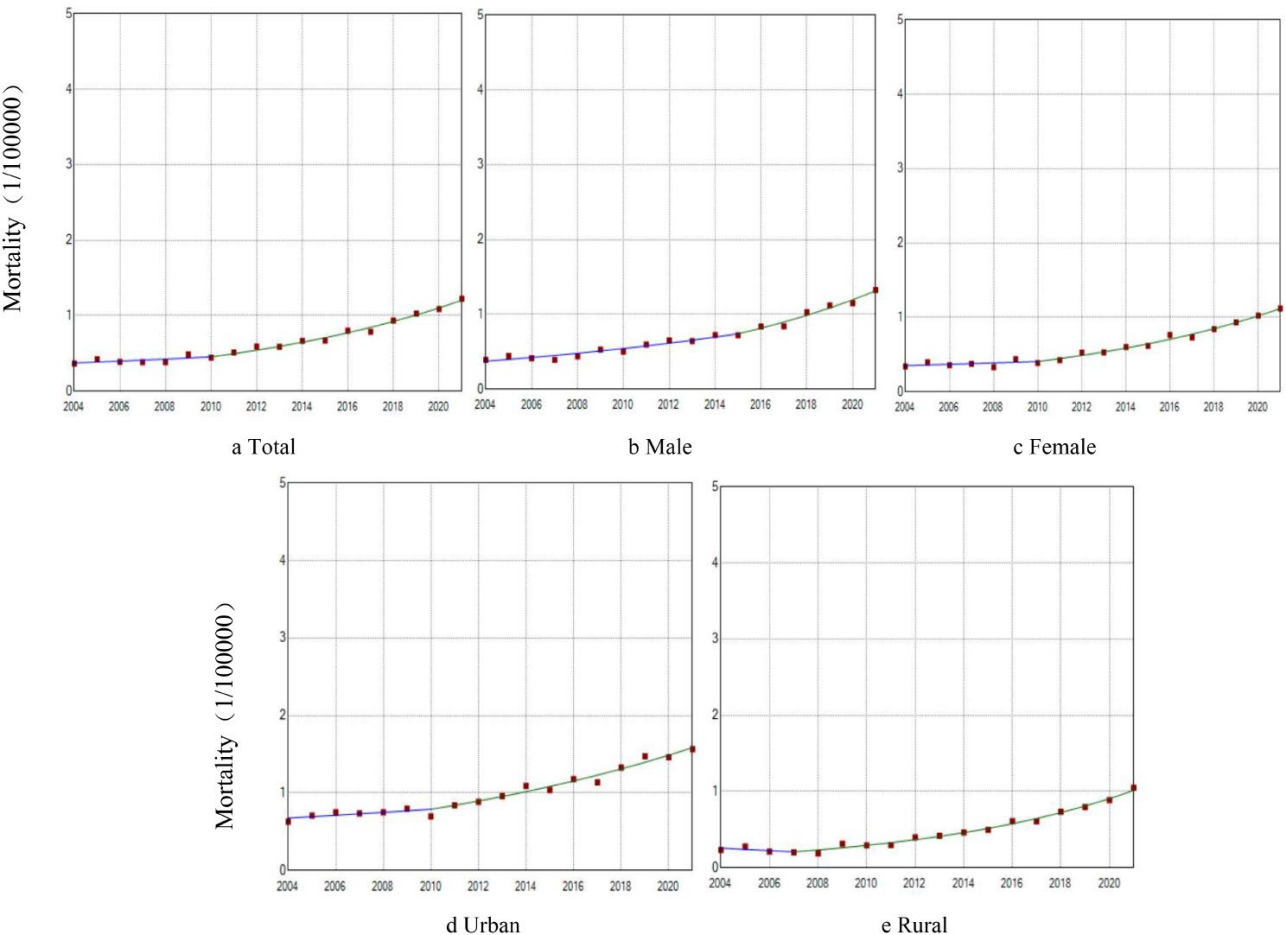
Trends in PD morality rates from 2004 to 2021 stratified by sex and urban/rural areas (per 100,000 population) (**a**) Total: 2004－2010: APC = 3.33; 2010－2021: APC = 9.27*; AAPC = 7.14*. (**b**) Male: 2004－2015: APC = 6.38*; 2015－2021: APC = 10.02*; AAPC = 7.65*. (**c**) Female: 2004－2010: APC = 2.46; 2010－2021: APC = 9.62*; AAPC = 7.03*. (**d**) Urban: 2004－2010: APC = 2.62; 2010－2021: APC = 6.53*; AAPC = 5.13*. (**e**) Rural: 2004－2007: APC=-6.78; 2007－2021: APC = 11.96*; AAPC = 8.40* *Note* *P < 0.05

**Fig. 2:**
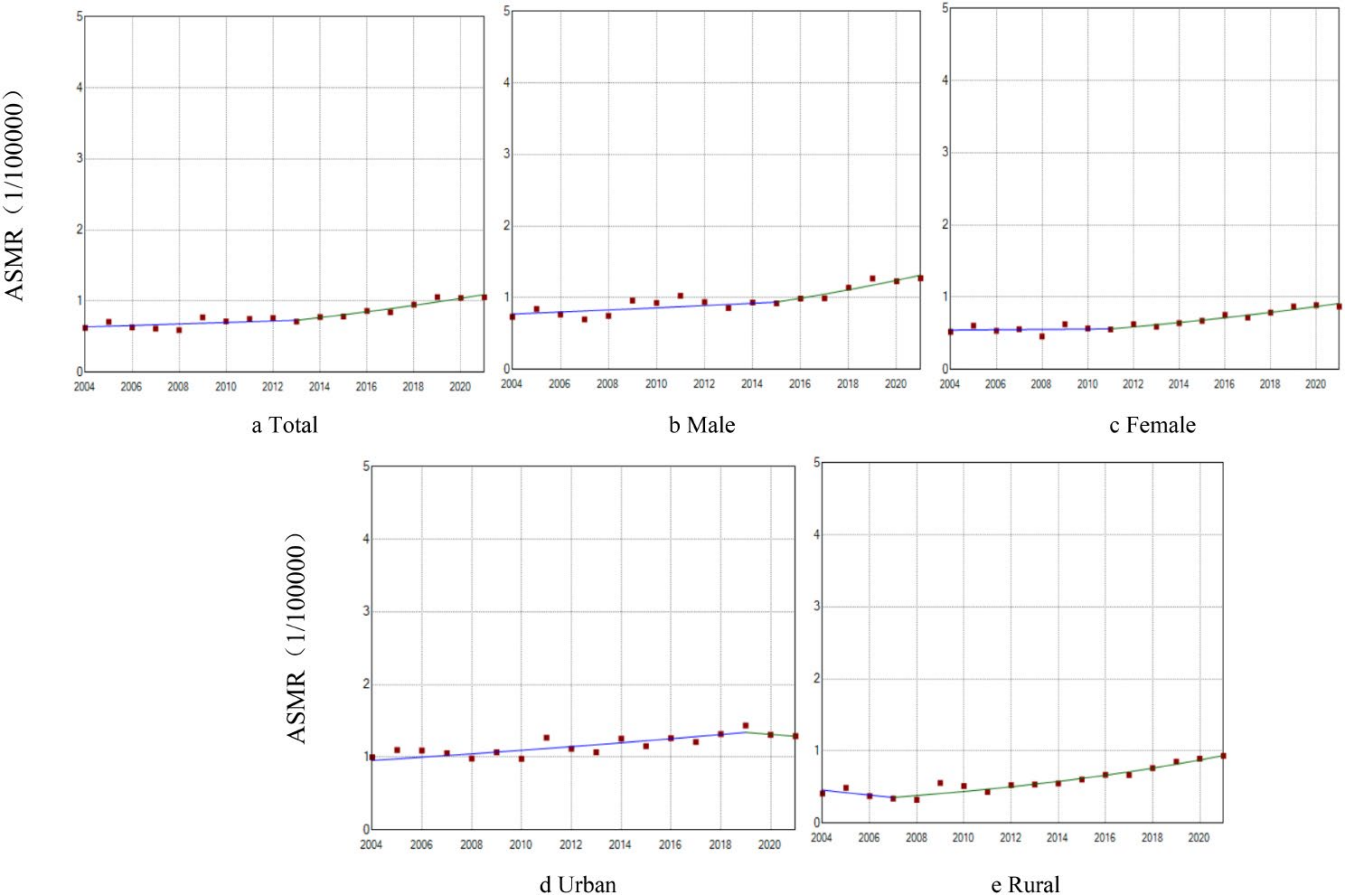
Trends in standardized morality rates of PD from 2004 to 2021 by sex and urban/rural (per 100,000 population). (**a**) Total: 2004－2013: APC = 1.51; 2013－2021: APC = 5.15*; AAPC = 3.21*. (**b**) Male: 2004－2015: APC = 1.79; 2015－2021: APC = 5.78*; AAPC = 3.18*. (**c**) Female: 2004－2011: APC = 0.41; 2011－2021: APC = 5.01*; AAPC = 3.09*. (**d**) Urban: 2004－2019: APC = 2.29*; 2019－2021: APC=-2.08; AAPC = 1.76*. (**e**) Rural: 2004－2007: APC=-8.01; 2007－2021: APC = 7.14*; AAPC = 4.29* *Note* *P < 0.05

The mortality (AAPC = 7.14%, *P* < 0.001) and standardized mortality rates of PD (AAPC = 3.21%, *P* < 0.001) among people living in China increased from 2004 to 2021. Both rates increased in both male and female populations (male: AAPC = 7.65%, AAPC_ASMR_=3.18%, *P* < 0.001; female: AAPC =7.03%, AAPC_ASMR_=3.09%, *P* < 0.001). The mortality and standardized mortality rates in male were higher than the female mortality rates (*P* < 0.001). From 2004 to 2021, the PD mortality and standardized mortality rates in both urban and rural areas showed an increasing trend (urban: AAPC = 5.13%, AAPC_ASMR_=1.76%, *P* < 0.001; rural: AAPC = 8.40%, AAPC_ASMR_=4.29%, *P* < 0.001). The urban mortality and standardized mortality rates were higher than those in rural areas (*P* < 0.001) (Figs. 1 and 2). In addition, our analysis of urban and rural areas by gender was consistent with the overall trend.

### Trends of PD mortality by age groups

**Fig. 3:**
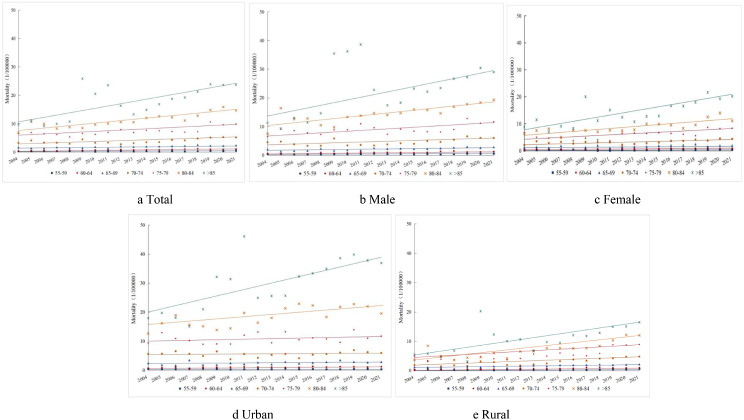
Trends in standardized morality of PD from 2004 to 2021 by age and sex/urban/rural (per 100,000 population). (**a**)Total: 55–59:APC = 3.05*;60–64:APC = 4.49*;65–69:APC = 3.28*;70–74:APC = 3.67*; 75–79: APC = 3.34*; 80–84: APC = 3.91*; >85: APC = 5.13*. (**b**)Male: 55–59:APC = 3.44*;60–64:APC = 4.94*;65–69:APC = 3.46*;70–74:APC = 3.66*; 75–79: APC = 2.85*; 80–84: APC = 3.35*; >85: APC = 4.13*. (**c**)Female: 55–59:APC = 2.08;60–64:APC = 3.36*;65–69:APC = 2.78*;70–74:APC = 3.59; 75–79: APC = 3.68*; 80–84: APC = 4.45*; >85: APC = 5.69*. (**d**)Urban: 55–59:APC=-0.65;60–64:APC = 1.87*;65–69:APC = 1.99*;70–74:APC = 1.12; 75–79: APC = 0.79; 80–84: APC = 2.07*; >85: APC = 4.18*. (**e**)Rural: 55–59:APC = 5.55*;60–64:APC = 6.53*;65–69:APC = 4.29*;70–74: APC = 6.55*; 75–79: APC = 6.56*; 80–84: APC = 6.68*; >85: APC = 6.51* *Note* *P < 0.05

The mortality of PD showed an increasing trend in the 55–59, 60–64, 65–69, 70–74, 75–79, 80-84 and > 85 age groups (APC was 3.05%, 4.49%, 3.28%, 3.67%, 3.34%, 3.91% and 5.13%, respectively;*P* < 0.05). Moreover, the mortality rate of PD increased with the increase of age, and the mortality rate of PD was the highest in the > 85 age group. Among male, PD mortality showed an upward trend in all age groups (APC was 3.44%, 4.94%, 3.46%, 3.66%, 2.85%, 3.35% and 4.13%, respectively; *P* < 0.05), and the mortality rate in the 60–64 age group showed the fastest trend (APC = 4.94%, *P* < 0.001). Among female, PD mortality showed an increase in all age groups except 55–59 age groups (APC was 3.36%, 2.78%, 3.59%, 3.68%, 4.45% and 5.69%, respectively; *P* < 0.05), the mortality rate in the > 85 age group showed the fastest trend (APC = 5.69%, *P* < 0.001). Among urban residents, PD mortality was on the rise in age groups 60–64, 65–69, 80–84 and > 85 (APC was 1.87%, 1.99%, 2.07% and 4.18%, respectively; *P* < 0.05), the mortality rate in the > 85 age group showed the fastest trend (APC = 4.18%, *P* < 0.001). For rural areas, PD mortality was on the rise in all age groups (APC was 5.55%, 6.53%, 4.29%, 6.55%, 6.56%, 6.68% and 6.51%, respectively; *P* < 0.05), the mortality rate in the 80–84 age group showed the fastest trend (APC = 6.68%, *P* < 0.001). Owing to the number of deaths in the 30–34, 35–39, 40–44, 45–49 and 50–54 age groups being mostly 0, the Joinpoint software could not conduct a trend analysis (Fig. 3).

## Discussion

Our study described the trend of PD mortality between 2004 and 2021 among Chinese residents in order to provide targeted intervention, improve the quality of life of PD in China, and evidence-based recommendations for public health decision-making and health resource management.

The mortality rate of PD and age-adjusted mortality rate of Chinese residents increased from 2004 to 2021, consistent with findings from several other studies [10, 14]. The increased mortality associated with PD is significantly associated with multiple risk factors including air pollution, pesticide (insecticide) exposure, type 2 diabetes, poor diet, and physical inactivity. A study conducted in the United States showed that organic matter and nitrates in particulate matter with a diameter ≤ 2.5 μm (PM_2.5_) were positively associated with worsening PD [17]. Exposure to nitric oxide was associated with an increased risk of PD (the risk ratio between the highest and lowest quartiles was 1.41; 95% CI: 1.02–1.95; *P* = 0.045) [18]. Entry of pesticides into the human body has been shown to trigger PD pathogenesis, and exposure to paraquat increases the risk of PD by 150% [19]. Duration of diabetes increased mortality among patients who developed T2D before PD onset [20]. Dairy products had a uric acid-lowering effect, and the intake of low-fat dairy milk along with high cholesterol levels can also increase the risk of PD [21]. However, healthy dietary patterns that include the high intake of vegetables, fruit, polyunsaturated fatty acids, whole grains, and nuts, reduce non-motor symptoms that precede PD diagnosis [22]. Previous studies have shown that physical inactivity worsens depression in patients with PD [23] and that regular physical activity such as aerobic exercise reduces the risk of PD [24].The results of our study showed the inflection point of the overall standardized mortality rate of PD in China was 2013, and the standardized mortality rate increased significantly from 2013 to 2021, which might be due to the use of adjusted monitoring point data since the national cause of death surveillance system began in 2013.

We found that the mortality rate of PD was higher in male than in female, which was in accord with the results of other scholars. A study of PD death trends in the United States between 1990 and 2019 showed the age-adjusted death rate for male was twice that for female [10]. Another study on PD mortality conducted in Italy between 1980 and 2015 also confirmed a higher mortality rate in male than in female [25]. Compared to female, male are more susceptible to PD, which is four times as prevalent in male than in female [26]; this may be a result of differences in physiological and clinical characteristics. For example, estrogen has a neuroprotective effect, and lower estrogen levels throughout life increase the risk of PD in male [27]. The PD phenotype is more severe in male residents at the time of onset, with female showing more signs of tremor and male showing more bradykinesia or stiffness [28]. Moreover, the overall cognitive and language deficit status of males with PD is greater than that of female patients with PD [29]. One study suggested that male residents were more likely to experience head trauma and exposure to toxic substances [30]. Several previous studies have identified a PD susceptibility gene on the X chromosome, explaining the higher incidence in male [31]. Thus, we recommend prioritizing early identification and preventive measures in male, strengthening health publicity and education. The government should strengthen occupational health protection, improve personal protection measures against exposure to toxic substances such as pesticides and chemicals, research and develop technical equipment to prevent toxic substances. In addition, health promotion and education should be promoted and strengthened, calling for proper physical exercise and the development of good living habits, such as the intake of a diversified and balanced diet, eating more fresh vegetables, foods rich in dietary fiber, and ensuring adequate protein intake.

The PD mortality rate in this study was higher in urban residents than in rural areas, consistent with previous findings in Taiwan [32], Italy [33], and Estonia [34]. This may be a result of the unequal distribution of medical resources, lack of awareness of PD, unequal access to health care, and environmental and lifestyle differences. Most of China’s high-quality hospitals are located in urban areas with more medical resources, and residents in urban areas have better medical care than those in rural areas; thus, urban areas have a higher detection rate, and PD is detected earlier in these patients [35]. Rural residents have insufficient knowledge of PD owing to a lack of higher education; consideration of PD symptoms as a common aging phenomenon; and limited access to specialist care, primary health care, and specialist services [36]. Older patients with PD may struggle to afford expensive drug treatments; moreover, rural residents have limited hospital access. Prior research has shown that the high incidence of PD in urban populations may be related to environmental and lifestyle factors [37]. With the acceleration of urbanization, people living in cities experience inflammatory changes in their brains due to high levels of air pollution in contrast to people living in rural areas [38]. Therefore, we suggest that the Chinese government strengthen the early screening, intervention, and classification management of PD to improve the treatment rate of urban PD. The government should take measures to control urban environmental pollution, reduce pollutant emissions from the source and exposure to dangerous environments. Furthermore, the publicity and education of PD risk factors should be improved, while advocating a healthy lifestyle. In addition, the government should increase investment in medical resources in rural to improve the efficiency of medical resource allocation, such as providing high-end medical equipment and experienced PD doctors to increase PD detection rates. Finally, the government should improve the ability of medical staff in primary medical and health institutions to diagnose and treat PD, and provide standardized basic medical services for PD.

The age group analysis in this study indicated that older people had a greater chance of dying from PD, mortality rates increased with age, and most deaths occurred at 85 years older, in agreement with previous studies [39–40]. Age was the most important determinant of the risk of death from PD, and the age-related risk of death in patients with PD was twice that in the general population [41]. Dopaminergic neurons become more vulnerable with age [42], and older people have a higher risk of developing comorbidities such as dementia, heart failure, and osteoporosis [43]. An increased incidence of PD may lead to increased mortality among the aging population, and longer life expectancies worldwide also increase the likelihood that people will be diagnosed with PD [4]. In 2020, there were 2.64 million people over the age of 65 in China, 13.5% of whom were over 80 years of age. Longevity may increase the number of patients with advanced PD who are more difficult to treat and often have less access to adequate healthcare [6]. Therefore, in the face of the increasingly aging population, the Chinese government should actively promote the establishment of an integrated medical and nursing service system for PD prevention, diagnosis, treatment and rehabilitation for the elderly, realize the organic integration of medical treatment, nursing care, and improve the medical services for elderly and improve their quality of life. Additionally, they should implement PD prevention and treatment actions, promote PD screening and intervention in the elderly, and provide early screening, diagnosis, and intervention for the elderly with potential PD. Moreover, the government should encourage technicians to develop intelligent auxiliary devices for elderly with PD to improve its cure rate. The government should also adjust resource allocation and encourage primary medical and health institutions to provide home medical and care services for elderly with PD. In addition, the elderly with PD should be encouraged to regularly participate in rehabilitation training, such as Baduanjin, Tai Chi and other traditional physical exercises to improve motor functions.

An interesting finding of this study was that the mortality rate of female changed faster than that of male in different age groups, with the highest increase in female aged > 85 years. Studies had shown that the longer life expectancy of female may explain the higher proportion of PD among female aged > 85 years [16].In addition, in postmenopausal female, the diminished protective effect of estrogen increases the risk of PD [44]. Compared with male, female tend to have faster disease progression and are less likely to receive less informal care support [45–46]. Our study also found the changing trend of mortality in rural was faster than that in urban among different age groups, with the highest increase in mortality in rural aged 80–84 years. This may be because elderly with PD often suffer from multiple diseases. And compared with urban, medical care was weaker and the accessibility to medical services was inadequate in rural [47]. In addition, due to the lack of professional psychiatrists, the elderly people need to travel long distances to obtain treatment. It is recommended that healthcare professionals focus on screening female aged >85 years to control risk factors by making appropriate lifestyle adjustments. The government should increase policy and resource support for rural PD, strengthen the understanding of primary medical staff on the clinical manifestations, prevention, treatment and rehabilitation of PD to improve the level of clinical diagnosis. Increasing residents’ awareness of PD and effective therapeutic drugs, high-risk groups should be monitored regularly. And exercise should be encouraged as it is important to prevent the delay and onset of PD.

### Limitations

The current study had certain limitations that might have affected the accuracy of our results. First, the connection-point regression model can only describe the changing trend of PD mortality, but cannot explain the factors affecting PD mortality. Further research is necessary to elucidate the causes of these different trends. Second, our study did not examine the association between influencing factors and PD. This is the next step for our team to work towards.

## Conclusion

In conclusion, we found that the mortality and standardized mortality rates for PD in China rose between 2004 and 2021, with higher PD mortality rates in male than in female and in urban areas than in rural areas. Additionally, older people were found to be more susceptible to death from PD, primarily at > 85 age group. Therefore, more attention should be paid to male, urban residents, and aged > 85 years to understand the risk factors of PD and improve quality of life in people with PD.

## Data Availability

All data generated or analyzed during this study are included in this published article.

## Abbreviations

PD: Parkinson’s disease
APC: annual percentage change
AAPC: average annual percentage change
DSP: Disease Surveillance Points
ASMR: age-standardized mortality rate
